# Effects of pegylated recombinant human granulocyte colony-stimulating factor on lymphocytes and white blood cells of patients with malignant tumor

**DOI:** 10.1515/biol-2022-0590

**Published:** 2023-04-12

**Authors:** Tong Zhao, Yuejun Wang, Deqing Zhou, Weike Zhang

**Affiliations:** Department of Oncology, The Eighth People’s Hospital of Jinan, No. 68, Xinxing Road, Gangcheng District, Jinan 271104, P.R. China; Department of Medical Administration, Jinan Gangcheng District Health Bureau, Jinan 270016, P.R. China; School of Finance, Central University of Finance and Economics, Haidian District, Beijing 100081, P.R. China

**Keywords:** pegylated recombinant human granulocyte colony stimulating factor, malignant tumor, lymphocyte, white blood cell

## Abstract

We investigated the effect of pegylated recombinant human granulocyte colony-stimulating factor (PEG-rhG-CSF) on lymphocytes and white blood cells of patients with malignant tumors. After PEG-rhG-CSF treatment, the count of lymphocytes increased in 66 cases, remained unchanged in 2 cases, and decreased in 20 cases. The difference in lymphocyte count before and after treatment was statistically significant (*P* < 0.001). White blood cell changes were positively correlated with lymphocyte changes (*r* = 0.36, *P* = 0.001). In the subgroup with increased white blood cells (*n* = 80), there were 62 cases with increased lymphocytes, 1 case with unchanged lymphocytes, and 17 cases with decreased lymphocytes after PEG-rhG-CSF treatment. There was significant difference in the count of lymphocytes and white blood cells (*P* < 0.001). In the subgroup with 6 mg of PEG-rhG-CSF (*n* = 66) and the subgroup with 3 mg of PEG-rhG-CSF (*n* = 22), the changes of white blood cell and lymphocyte counts before and after treatment were statistically significant (*P* < 0.001). The two were positively correlated in the 6 mg PEG-rhG-CSF subgroup, with correlation coefficient *r* = 0.34 (*P* = 0.002). PEG-rhG-CSF can increase the count of lymphocytes and white blood cells in patients with malignant tumors, and the increase of lymphocytes is positively correlated with the increase of white blood cells.

## 1 Introduction

Chemotherapy and radiotherapy are still important modes of comprehensive treatment for malignant tumors [1], and neutropenia is the most common hematological toxicity during chemotherapy and radiotherapy. It often induces fever during neutropenia (FN), septic shock, and even death, which seriously affects the clinical treatment and survival of tumor patients and increases medical expenses [2,3]. Pegylated recombinant human granulocyte colony-stimulating factor (PEG-rhG-CSF) is a glycoprotein that can bind to specific receptors on the surface of granulocyte progenitor cells or mature neutrophils. It can promote the proliferation and differentiation of granulocyte progenitor cells and enhance the phagocytosis and killing ability of neutrophils [4]. PEG-rhG-CSF is a long-acting, self-regulating stimulator with a plasma half-life of 47 h and only needs to be applied once after each chemotherapy cycle. The standardized management guidelines for chemotherapy-related neutropenia have been issued [5,6], which recommends the prophylactic application of PEG-rhG-CSF at 24–72 h after chemotherapy. PEG-rhG-CSF can effectively reduce the incidence and severity of neutropenia caused by chemotherapy and shorten the duration of neutropenia [7,8]. However, the effects of PEG-rhG-CSF on lymphocytes and white blood cells remain unclear.

Herein, this study analyzed the effect of PEG-rhG-CSF on lymphocyte and white blood cell count during chemotherapy in patients with malignant tumors.

## 2 Materials and methods

### 2.1 Participants

This is a retrospective observational study. Initially, 183 patients who received PEG-rhG-CSF in the Department of Oncology of the Eighth People’s Hospital of Jinan from January 2021 to June 2022 were screened. Finally, 88 patients with nonhematologic malignant tumors were enrolled in this study. The inclusion criteria were as follows: 1) patients with histopathologically or cytologically confirmed nonhematologic malignant tumors; 2) patients with age equal to or greater than 18 years; 3) patients with Eastern Cooperative Oncology Group Performance Status (ECOG-PS) score less than or equal to 2; 4) before chemotherapy, white blood cells ≥4.0 × 10^9^/L, absolute neutrophil count ≥2.0 × 10^9^/L, hemoglobin ≥9.0 g/dL, and platelets ≥80 × 10^9^/L; 5) patients with normal coagulation function; and 6) patients with normal heart, liver, and kidney function. The exclusion criteria were: 1) patients with repeated myelosuppression in previous chemotherapy; 2) patients with uncontrollable infection; 3) patients with previous hematopoietic stem-cell transplantation or organ transplantation; 4) patients with previous radiotherapy treatment; 5) patients with bone marrow invasion; 6) patients with kidney metastasis, adrenal metastasis, or abnormal renal function, such as abnormal creatinine clearance rate and blood urea nitrogen; 7) patients with other serious complications; and 8) patients with hematologic system disease.

#### 2.1.1 Sample size calculation

The sample size was calculated using the following formula:

n=\frac{{({Z}_{\alpha }+{Z}_{\beta })}^{2}\times \overline{\rho }(1-\overline{\rho })}{{({\rho }_{1}-{\rho }_{0})}^{2}},]

where *α* = 0.05, 1 – *β* = 0.90, {Z}_{\alpha }] = 1.64, {Z}_{\beta }] = 1.28, \overline{\rho }=\frac{{\rho }_{1}+{\rho }_{0}}{2}] , and {\rho }_{1}=\frac{{\rho }_{0}\text{OR}}{1+{\rho }_{0}(\text{OR}-1)}] . According to the pilot experiment, *ρ*_0_ was determined as 0.2 and OR was determined as 3.079. The calculated sample size was 82. Finally, 88 cases were enrolled in this study.


**Informed consent:** Informed consent has been obtained from all individuals included in this study.
**Ethical approval:** The research related to human use has been complied with all the relevant national regulations, institutional policies, and in accordance with the tenets of the Helsinki Declaration, and has been approved by the Ethics Committee of the Eighth People s Hospital of Jinan (No. 2020053). This study has been registered at Chinese Clinical Trial Register (ChiCTR2200063211).

### 2.2 Medication

For primary or secondary prevention of chemotherapy-related neutropenia, patients were subcutaneously injected with PEG-rhG-CSF (6 or 3 mg; Qilu Pharmaceutical Co., Ltd, Jinan, China; specification: 3 mg × 1 mL/piece, s20150013) on day 3 after chemotherapy.

### 2.3 Blood routine test

According to the pharmacokinetic parameters of PEG-rhG-CSF [9], we chose 4–7 days after injection of PEG-rhG-CSF as the observation time point. All patients received blood routine tests on days 3 and 7–10 after chemotherapy to evaluate the changes of lymphocytes. Blood routine test was performed using BC-5180 Automatic Blood Cell Analyzer (Mindray Biomedical Electronics Co., Ltd., Shenzhen, China).

### 2.4 Data collection

Clinical data were collected including 1) demographics (sex and age), 2) pathological diagnosis, 3) ECOG PS score, and 4) blood routine results.

### 2.5 Statistical analysis

SPSS 19.0 statistical software was used. The measurement data were expressed as mean ± standard deviation, and compared with paired sample *t*-test. Pearson regression analysis was used for correlation analysis. *P* < 0.05 was considered as statistically significant.

## 3 Results

### 3.1 Baseline characteristics of patients

The baseline characteristics of patients are shown in Table 1. Of 88 patients, 66 cases received 6 mg of PEG-rhG-CSF and 22 cases received 3 mg of PEG-rhG-CSF after chemotherapy. There were 41 cases of nonsmall cell lung cancer, 11 cases of small cell lung cancer, 14 cases of breast cancer, 6 cases of esophageal cancer, 11 cases of colon cancer, and 5 cases of gastric cancer. Their mean age was 49.37 ± 9.53 years. There were 47 male and 41 female patients. Additionally, 84 cases had an ECOG PS score of 0–1 and 4 cases had an ECOG PS score of 2.

**Table 1:** Baseline characteristics of patients

Characteristics	All patients (*N* = 88)	PEG-rhG-CSF 6 mg group (*N* = 66)	PEG-rhG-CSF 3 mg group (*N* = 22)
Median age – years old	49.37 ± 9.53	49.23 ± 6.23	49.44 ± 5.48
**Sex – No. (%)**			
Male	47 (53.41)	35 (53.03)	12 (54.55)
Female	41 (46.59)	31 (46.97)	10 (45.45)
**Tumor type – No. (%)**			
Nonsmall cell lung cancer	41 (46.59)	32 (48.48)	9 (40.90)
Small cell lung cancer	11 (12.50)	8 (12.12)	3 (13.64)
Breast cancer	14 (15.91)	10 (15.15)	4 (18.18)
Esophageal cancer	6 (6.82)	5 (7.58)	1 (4.55)
Colon cancer	11 (12.50)	9 (13.64)	2 (9.09)
Gastric cancer	5 (5.68)	2 (3.03)	3 (13.64)
ECOG PS score: 0, 1 No. (%)	84 (95.45)	63 (95.45)	21 (95.45)
ECOG PS score: 2 No. (%)	4 (4.55)	3 (4.55)	1 (4.55)

### 3.2 Comparison of white blood cell and lymphocyte counts before and after treatment with PEG-rhG-CSF

In the 88 patients, there were 80 cases with increased white blood cells and 8 cases with decreased white blood cells after treatment with PEG-rhG-CSF. There were 66 cases with increased lymphocytes, 2 cases with no significant change in lymphocytes, and 20 cases with decreased lymphocytes. Statistically, the white blood cell and lymphocyte counts after treatment with PEG-rhG-CSF were significantly higher than those of before treatment (*P* < 0.001) (Table 2).

**Table 2:** Comparison of white blood cell count and lymphocyte count before and after PEG-rhG-CSF treatment

Variable (×10^9^/L)	Mean ± standard deviation	*t*	*P*	Difference 95% CI
White blood cell count before PEG-rhG-CSF treatment	5.69 ± 2.84	−10.91	<0.001	−19.20 to −13.28
White blood cell count after PEG-rhG-CSF treatment	21.93 ± 14.02			
Lymphocyte count before PEG-rhG-CSF treatment	1.22 ± 0.45	−6.37	<0.001	−0.70 to −0.37
Lymphocyte count after PEG-rhG-CSF treatment	1.75 ± 0.90			

### 3.3 Correlation between the white blood cell count change and lymphocyte change before and after treatment with PEG-rhG-CSF

**Figure 1:**
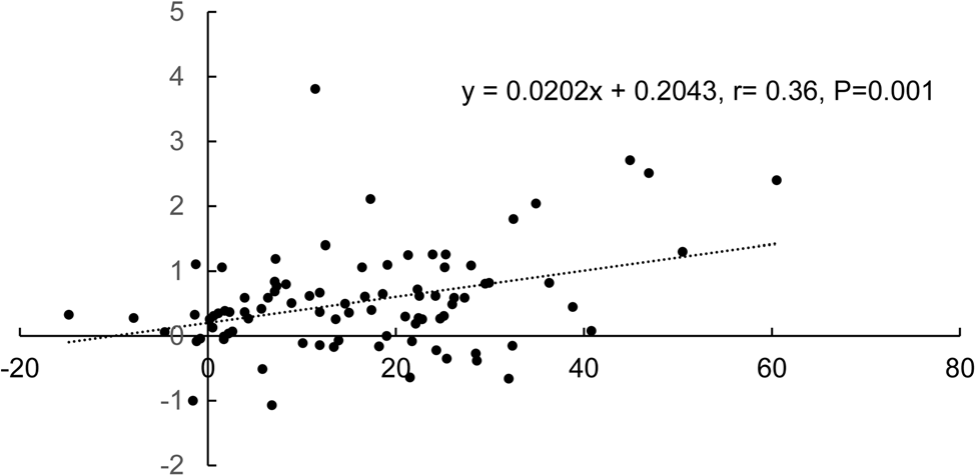
Scatter plot of correlation analysis between lymphocyte change and white blood cell change in all patients (*n* = 88).

Pearson regression analysis was conducted with lymphocyte change (*x*) as the explained variable and white blood cell change (*y*) as the explanatory variable: *y* = 0.0202*x + 0.2043 (Figure 1). The results showed that white blood cell change was positively correlated with lymphocyte change in all 88 patients, with a correlation coefficient of 0.36 (*P* = 0.001). The regression coefficient was highly significant (1% significance level), which indicates that lymphocyte change showed a significant linear relationship with white blood cell change. On average, for every 1 × 10^9^/L increase in white blood cells, lymphocytes would increase by 0.02 × 10^9^/L.

### 3.4 Changes in lymphocyte count in the subgroup with increased white blood cells after treatment with PEG-rhG-CSF

Among the 80 cases with increased white blood cells after treatment with PEG-rhG-CSF, 62 cases had increased lymphocytes, 1 case had no significant change in lymphocytes, and 17 cases had decreased lymphocytes. The difference in lymphocyte count was statistically significant before and after PEG-rhG-CSF in the subgroup with increased white blood cells after treatment (*P* < 0.001), as shown in Table 3.

**Table 3:** Comparison of lymphocyte counts before and after PEG-rhG-CSF treatment in the subgroup with increased white blood cells

Variable (×10^9^/L)	Mean ± standard deviation	*t*	*P*	Difference 95% CI
Lymphocyte count before PEG-rhG-CSF treatment	1.22 ± 0.44	−6.47	<0.001	−0.75 to −0.40
Lymphocyte count after PEG-rhG-CSF treatment	1.79 ± 0.92			

### 3.5 Correlation between changes in lymphocyte count and white blood cell count in the subgroup with increased white blood cells after application of PEG-rhG-CSF treatment

**Figure 2:**
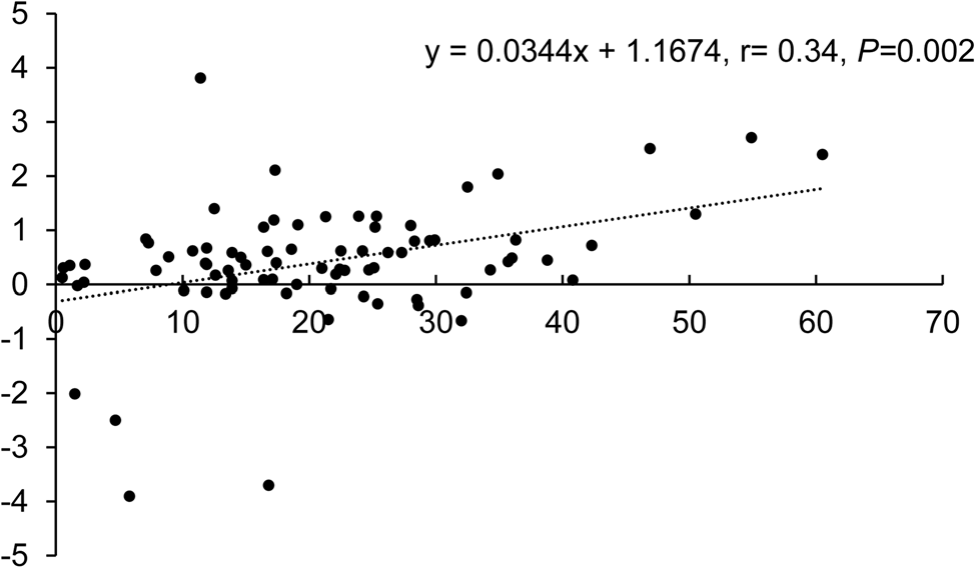
Scatter plot of correlation analysis between lymphocyte change and white blood cell change in the subgroup with increased white blood cells (*n* = 80).

In the subgroup with increased white blood cells after PEG-rhG-CSF treatment, Pearson regression analysis was performed with lymphocyte change (*x*) as the explained variable and white blood cell change (*y*) as the explanatory variable: *y* = 0.0344*x + 1.1674 (Figure 2). The results found that white blood cell change was positively correlated with lymphocyte change with a correlation coefficient of *r* = 0.34, *P* = 0.002. The regression coefficient was highly significant (1% significance level), which suggests a significant linear relationship between lymphocyte change and white blood cell change. For every 1 × 10^9^/L rise in white blood cells, lymphocytes will increase by 0.03 × 10^9^/L.

### 3.6 Analysis of subgroup that received 6 mg of PEG-rhG-CSF

A total of 66 patients were treated with 6 mg of PEG-rhG-CSF. As shown in Table 4, there were statistically significant changes in white blood cell count and lymphocyte count before and after treatment (*P* < 0.001). Similarly, in the subgroup with 6 mg of PEG-rhG-CSF, white blood cell count and lymphocyte count were positively correlated, with a correlation coefficient of *r* = 0.34 and *P* = 0.002.

**Table 4:** Comparison of white blood cell count and lymphocyte count before and after treatment in the 6 mg PEG-rhG-CSF subgroup

Variable (×10^9^/L)	Mean ± standard deviation	*t*	*P*	Difference 95% CI
White blood cell count before PEG-rhG-CSF treatment	5.95 ± 2.89	−10.46	<0.001	−22.59 to −15.35
White blood cell count after PEG-rhG-CSF treatment	24.92 ± 14.41			
Lymphocyte count before PEG-rhG-CSF treatment	1.26 ± 0.48	−5.74	<0.001	−0.77 to −0.37
Lymphocyte count after PEG-rhG-CSF treatment	1.83 ± 0.95			

### 3.7 Analysis of subgroup with 3 mg of PEG-rhG-CSF

A total of 22 patients were treated with 3 mg of PEG-rhG-CSF. There were also statistically significant changes in white blood cell count (*P* < 0.001) and lymphocytes (*P* = 0.012) before and after treatment, as shown in Table 5. However, there was no significant correlation between white blood cell count and lymphocyte count (*P* > 0.05).

**Table 5:** Comparison of white blood cell count and lymphocyte count before and after treatment in the 3 mg PEG-rhG-CSF subgroup

Variable (×10^9^/L)	Mean ± standard deviation	*t*	*P*	Difference 95% CI
White blood cell count before PEG-rhG-CSF treatment	4.92 ± 2.57	−5.81	<0.001	−10.93 to −5.17
White blood cell count after PEG-rhG-CSF treatment	12.97 ± 7.70			
Lymphocyte count before PEG-rhG-CSF treatment	1.09 ± 0.34	−2.76	0.012	−0.72 to −0.10
Lymphocyte count after PEG-rhG-CSF treatment	1.51 ± 0.67			

## 4 Discussion

Neutropenia occurs in 25–40% of patients treated initially with chemotherapy, and neutropenia results in delayed chemotherapy and dose reduction in more than 60% of patients. Human granulocyte CSF (hG-CSF) is often used to treat neutropenia in clinic. PEG-rhG-CSF has been confirmed to be as effective as hG-CSF in preventing and treating chemotherapy-related myelosuppression and FN [10]. However, hG-CSF is a short-acting drug and should be applied three to seven times per chemotherapy cycle. Meanwhile, PEG-rhG-CSF is a long-acting drug and needs to be applied only once at 24 h after each chemotherapy cycle, which is more convenient. In addition, Huang et al. showed that PEG-rhG-CSF had a stronger immune cell-modulating effect compared to rhG-CSF in breast cancer patients [11]. Therefore, we focused on PEG-rhG-CSF in this study.

PEG-rhG-CSF has a definite effect on granulocytes. However, there are limited studies on the effects of PEG-rhG-CSF on immune cells (especially lymphocytes), immune microenvironment, and immune status in patients with malignant tumors. This study investigated the effect of PEG-rhG-CSF on lymphocytes and white blood cells in patients with malignant tumors. Our results found that lymphocyte counts were significantly increased in 66 cases after PEG-rhG-CSF treatment. The white blood cell increase was positively correlated with lymphocyte increase (correlation coefficient *r* = 0.36, *P* = 0.001). Pearson regression analysis showed a highly significant regression coefficient (1% significance level). On average, for every 1 × 10^9^/L increase in white blood cells, lymphocytes will increase by 0.02 × 10^9^/L. This indicates that PEG-rhG-CSF can increase lymphocyte counts in patients with malignant tumors. Single-cell analysis by Franzke et al. showed that CD4 + T cells and CD8 + T cells also expressed G-CSF receptors after stimulation with short-acting G-CSF, and that G-CSF could regulate T cells directly through these receptors [12]. We speculate that it may be possible that treatment with long-acting PEG-rhG-CSF can also cause CD4 + T cells and CD8 + T cells in patients with malignancies to express G-CSF receptors, which in turn can regulate T cells and elevate lymphocyte counts through these receptors. More in-depth studies are needed to confirm this speculation.

In this study, we further analyzed the subgroup with increased white blood cells after PEG-rhG-CSF treatment. The changes in lymphocytes and white blood cells were also significantly different in this subgroup, and there was a positive correlation between increased white blood cells and increased lymphocytes, with a correlation coefficient of 0.34. A mean increase of 1 × 10^9^/L in white blood cells indicates a mean increase of 0.03 × 10^9^/L in lymphocytes.

In the subgroup with increased white blood cells after PEG-rhG-CSF treatment, the increase in lymphocytes was more significant along with the increase in white blood cells than in the all-case group. It is hypothesized that this may be because patients with increased white blood cells after PEG-rhG-CSF treatment may have higher expression of G-CSF receptors by lymphocyte CD4 + T cells and CD8 + T cells, which in turn may exert stronger effects on regulating T cells and elevating lymphocytes through G-CSF receptors.

Clinically, the commonly used dose of PEG-rhG-CSF for subcutaneous injection is 6 mg per cycle. If the body weight of patients is less than 45 kg or if the patients have a “white blood cell-like reaction” (white blood cells > 50 × 10^9^/L and neutrophils > 20 × 10^9^/L), the dose of PEG-rhG-CSF for subcutaneous injection can be adjusted to 3 mg per cycle [13]. In primary-care hospitals, PEG-rhG-CSF at 3 mg per cycle can also be used because of high cost of the drug. Therefore, this study analyzed the 6 mg group and the 3 mg group separately. The results showed that the 66 patients in the PEG-rhG-CSF 6 mg subgroup showed statistically significant changes in white blood cell count and lymphocyte count before and after treatment, and the two were positively correlated with a correlation coefficient of *r* = 0.34, *P* = 0.002. The 22 patients in the PEG-rhG-CSF 3 mg subgroup showed statistically significant changes in lymphocyte count and white blood cell count before and after treatment. However, there was no correlation between the changes in white blood cell count and lymphocyte count before and after treatment in the 3 mg subgroup. This may be caused by the small number of samples in the 3 mg subgroup. It should be further clarified in future studies by increasing the sample size.

In recent years, immunotherapy using programmed death factor-1 (PD-1) antibodies and chimeric receptor lymphocytes, for example, have been widely used as first-line class IA and recommended drugs for advanced lung cancer, esophageal cancer, and other solid tumors [14,15,16]. Despite longer overall survival, the overall response rate of immunotherapy is still low, only 10–20%, and the problem of primary and secondary drug resistance has been difficult to be solved [17,18].

There are many theories on the mechanism of poor efficacy and drug resistance of immunotherapy. Some scholars consider that the internal factors of tumor cells (such as activation of driver genes and inactivation of oncogenes) and external factors may lead to drug resistance [19]. In addition, there are other factors that affect the efficacy of immunotherapy, including the decrease of tumor-infiltrating lymphocytes, the low expression of PD-L1, and the decrease in absolute value of lymphocytes [20].

Long-term stimulation by tumor antigens can cause T cells to gradually lose their original ability to recognize antigens, activate and proliferate, or secrete interleukin-2, leading to T cell depletion [21]. The immune microenvironment and the distribution of immune cells in peripheral blood are important markers of immune status. A study has found that there were more CD3+, CD4+, CD8+, and PD-1+ T cells in the peripheral blood of lung adenocarcinoma patients with long-term survival, indicating that the infiltration degree of immune cells in the tumor immune microenvironment is closely related to long-term survival [22]. The distribution of peripheral blood lymphocyte subpopulations can be used as a prognostic indicator for tumor patients [23]. Understanding and improving the immune status of patients with malignant tumors, increasing the number of lymphocytes, reversing the depletion of lymphocytes, and changing the immune microenvironment are of great significance for improving the effect of immunotherapy.

In order to improve the efficacy of immunotherapy, some new combination immunotherapy models have been proposed, including immunotherapy combined with radiotherapy and double immunotherapy [24,25,26]. Our study confirmed that PEG-rhG-CSF could increase the number of lymphocytes in patients with malignant tumors. Immunotherapy combined with PEG-rhG-CSF may serve as a new mode of combined immunotherapy. On the one hand, PEG-rhG-CSF, as the primary or secondary prevention of agranulocytosis, can prevent agranulocytosis and FN and ensure smooth treatment. On the other hand, by increasing the number of lymphocytes, we may reverse the depletion of lymphocytes, improve the efficacy of immunotherapy, and reduce the occurrence of drug resistance.

The following were the limitations of this study: 1) although the sample size was large, the selective bias and recall bias of data selection were high; 2) the disease types of the study population were heterogeneous; 3) this study did not perform further detailed analysis of lymphocyte subgroups and did not conduct a T-cell antigen receptor group library study; and 4) follow-up study was not performed. Further prospective studies with specific tumor type and detailed analysis of lymphocyte subgroups (such as CD3+, CD4+, CD8+, NK cells, B cells, etc.) are needed.

## 5 Conclusion

In summary, PEG-rhG-CSF at both 3 and 6 mg per cycle can increase the count of lymphocytes and white blood cells in patients with malignant tumors. Immunotherapy combined with PEG-rhG-CSF may serve as a new combined immunotherapy mode to prevent granulocytopenia and FN, increase the number of lymphocytes in patients with malignant tumors, reverse lymphocyte depletion, and then improve the efficiency of immunotherapy.
